# Renal Schwannoma: Unraveling a Rare Tumor With Diagnostic Insights

**DOI:** 10.7759/cureus.84646

**Published:** 2025-05-22

**Authors:** Sai Swarupa Vulasala, Aryan Sharma, Connor Michalski, Abdullah Mohamed, Ashraf Abdulhalim, Dheeraj Gopireddy

**Affiliations:** 1 Radiology, University of Florida College of Medicine – Jacksonville, Jacksonville, USA; 2 Biology, Creekside High School, St. Johns, USA; 3 Diagnostic Radiology, University of Florida College of Medicine – Jacksonville, Jacksonville, USA; 4 Pathology and Laboratory Medicine, University of Florida College of Medicine – Jacksonville, Jacksonville, USA

**Keywords:** imaging, nerve sheath tumors, perinephric schwannoma, radiology, renal schwannoma

## Abstract

Schwannomas are rare nerve sheath tumors arising from Schwann cells. In general, schwannomas are more commonly seen in the head, neck, and extremities, along the cranial and peripheral nerves. Retroperitoneal schwannomas are rare and very few cases are reported in the literature. Here, we describe a case of renal schwannoma which presented as a progressive right renal mass and the diagnosis was confirmed histopathologically. The patient remained asymptomatic throughout the entire disease course, and the surveillance imaging aided in the early identification of the disease progression. In this case report, we detail the patient’s presentation, characteristic imaging findings, and histopathologic findings.

## Introduction

Schwannomas are benign nerve sheath tumors that originate from Schwann cells which insulate peripheral nerves. These can occur anywhere along the nerve course with high predilection for the head, neck, and extremities [1]. Visceral schwannomas are extremely rare, therefore making their diagnosis is challenging. Only about 1-3% of schwannomas are retroperitoneal, and less than 50 cases of renal schwannomas are reported in the literature [2-5]. Renal schwannomas have a slight female predominance and occur mostly in middle-aged adults [1]. Due to their typically slow growth and nonspecific presentation, they are often incidental findings during imaging studies for other conditions. This case report details a renal schwannoma identified as an incidental imaging finding and later confirmed with histopathology.

## Case presentation

**Figure 1:**
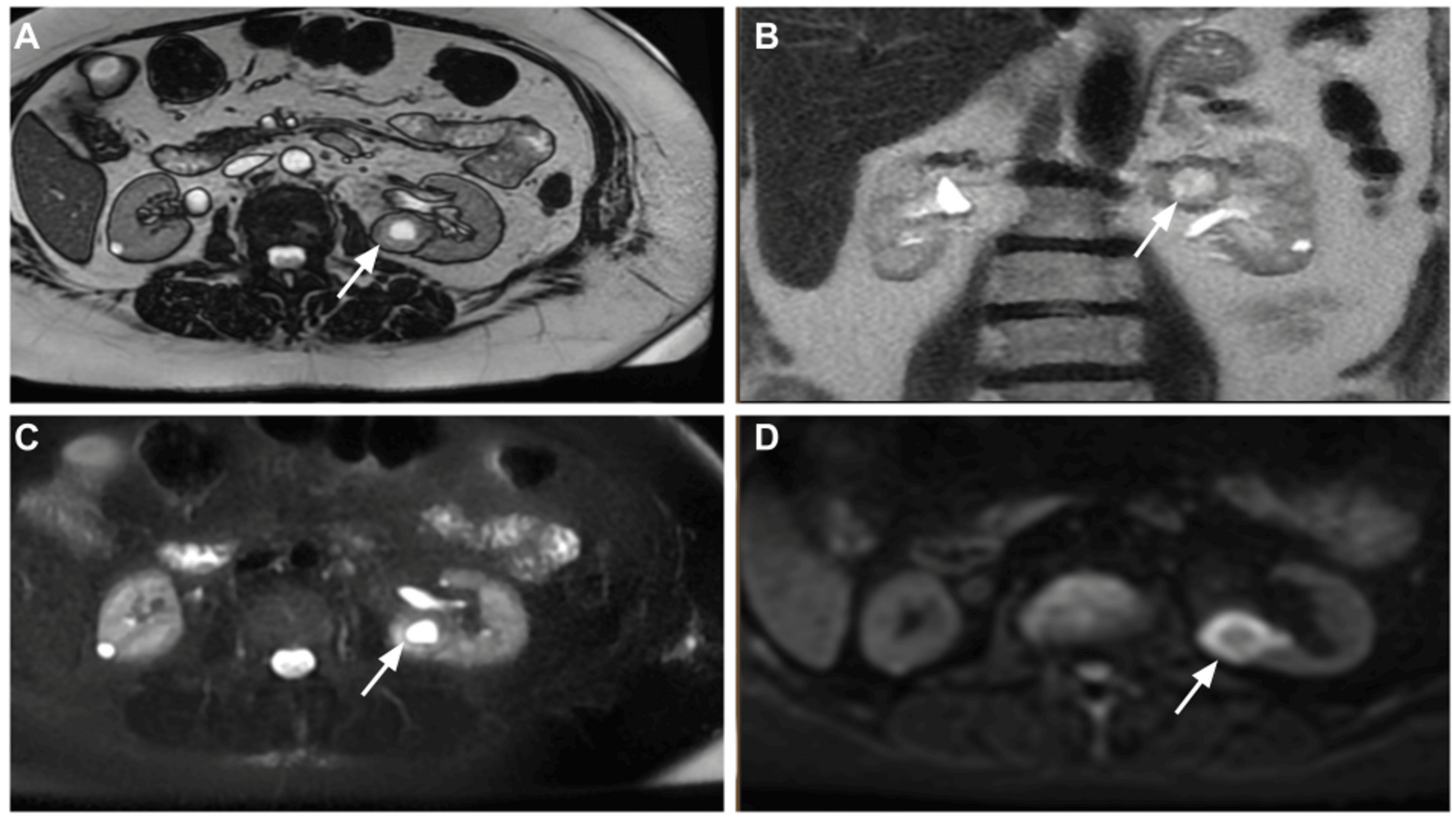
Magnetic resonance imaging of the abdomen on initial presentation revealed a lesion within in the left renal pelvis (white arrow) with absent microscopic fat on opposed phase images (A) and has central high signal intensity (white arrows) on coronal T2 non-fat saturated (B) and axial fat saturated STIR images (C). There is diffusion restriction on the peripheral aspect of the lesion (white arrow) on diffusion-weighted images (D). STIR: Short Tau Inversion Recovery.

A 73-year-old female patient with a medical history of transient ischemic attack, diabetes, hypertension, hyperlipidemia, steatotic liver disease, and gastric intestinal metaplasia was found to have an incidental left renal mass measuring up to 2.0 cm in maximal dimension in the posterior interpolar region on computed tomography of the abdomen. She denied a history of dysuria, hematuria, appetite loss, or weight loss. There was no family history of cancer or genetic disorders. She denied tobacco or alcohol abuse. On presentation, vitals were within normal limits. Blood pressure was 110/60 mmHg and heart rate was 78 beats per minute. Physical examination was unremarkable. Abdomen was nontender on palpation. There was no palpable abdominal mass. There was no evidence of skin rashes/lesions, neurologic deficits, or bone tenderness. Further imaging with MRI revealed a left renal pelvic lesion with benign characteristics as described in Figure 1 and a decision was made to follow up with surveillance CTs. This lesion remained stable on the follow-up surveillance CT for two years.

**Figure 2:**
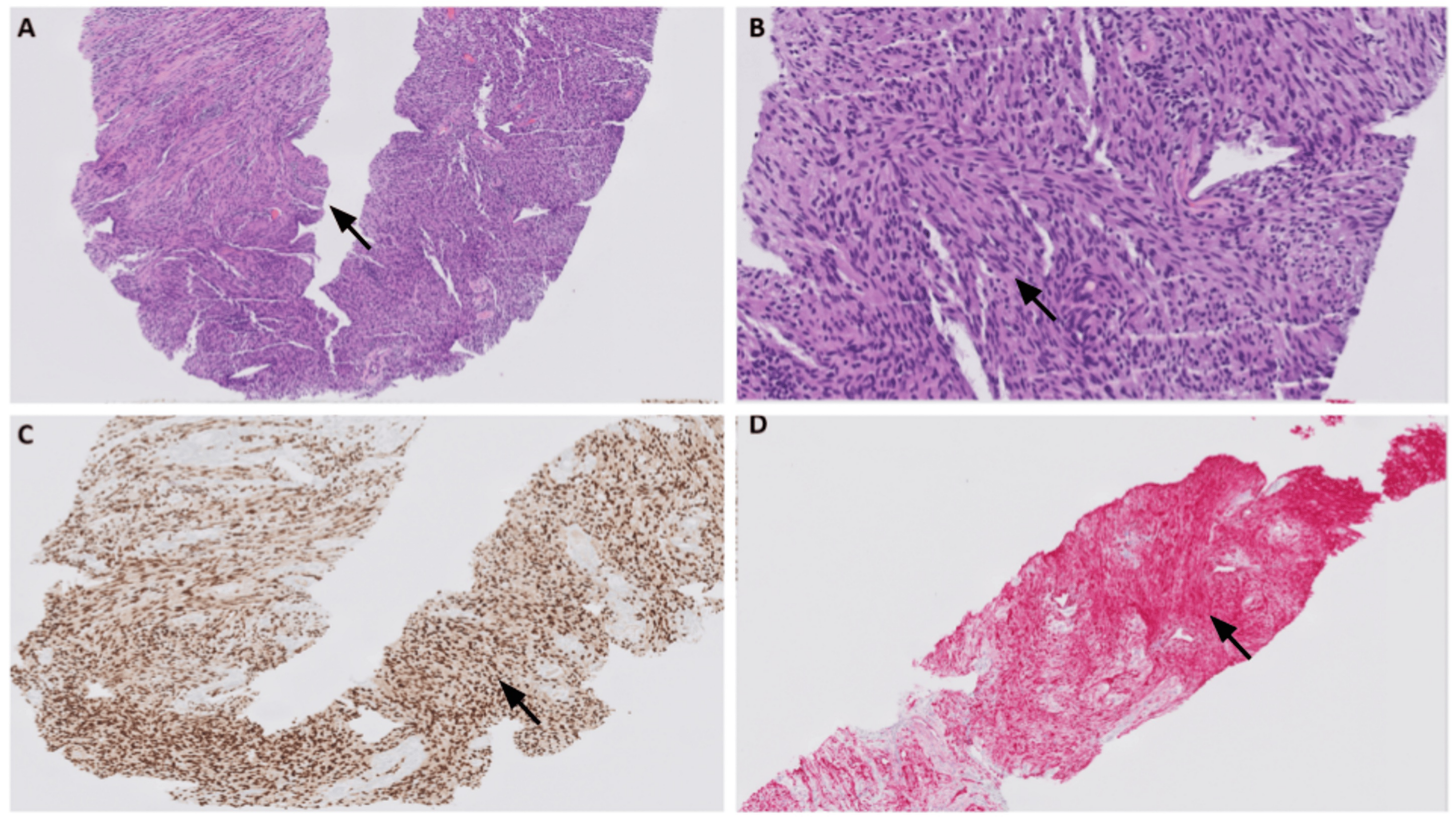
Microscopic images of hematoxylin and eosin (H&E) stained sections, SOX-10 and Red S100 immunohistochemical stains of schwannoma. (A) Low-power view (x5) showing a core biopsy of hypercellular spindle cell lesion with no hemorrhage or necrosis identified (black arrow). (B) High-power view (x20) displaying uniform (monomorphic) cells with narrow, elongated and wavy tapered ends with no overt features of malignancy (no mitosis or atypia identified) (black arrow). (C) SOX-10 immunohistochemical stain shows positive diffuse nuclear staining pattern (black arrow). (D) Red S-100 immunohistochemical stain shows positive diffuse nuclear and cytoplasmic staining pattern (black arrow).

The patient remained asymptomatic during this period. Surveillance CT three years later showed a stable left renal lesion and interval development of a new lesion adjacent to the prior one. CT-guided biopsy of the new lesion revealed spindle cells with no malignant features and stain positive for S-100, suggestive of schwannoma - Antoni A type (Figure 2).

**Figure 3:**
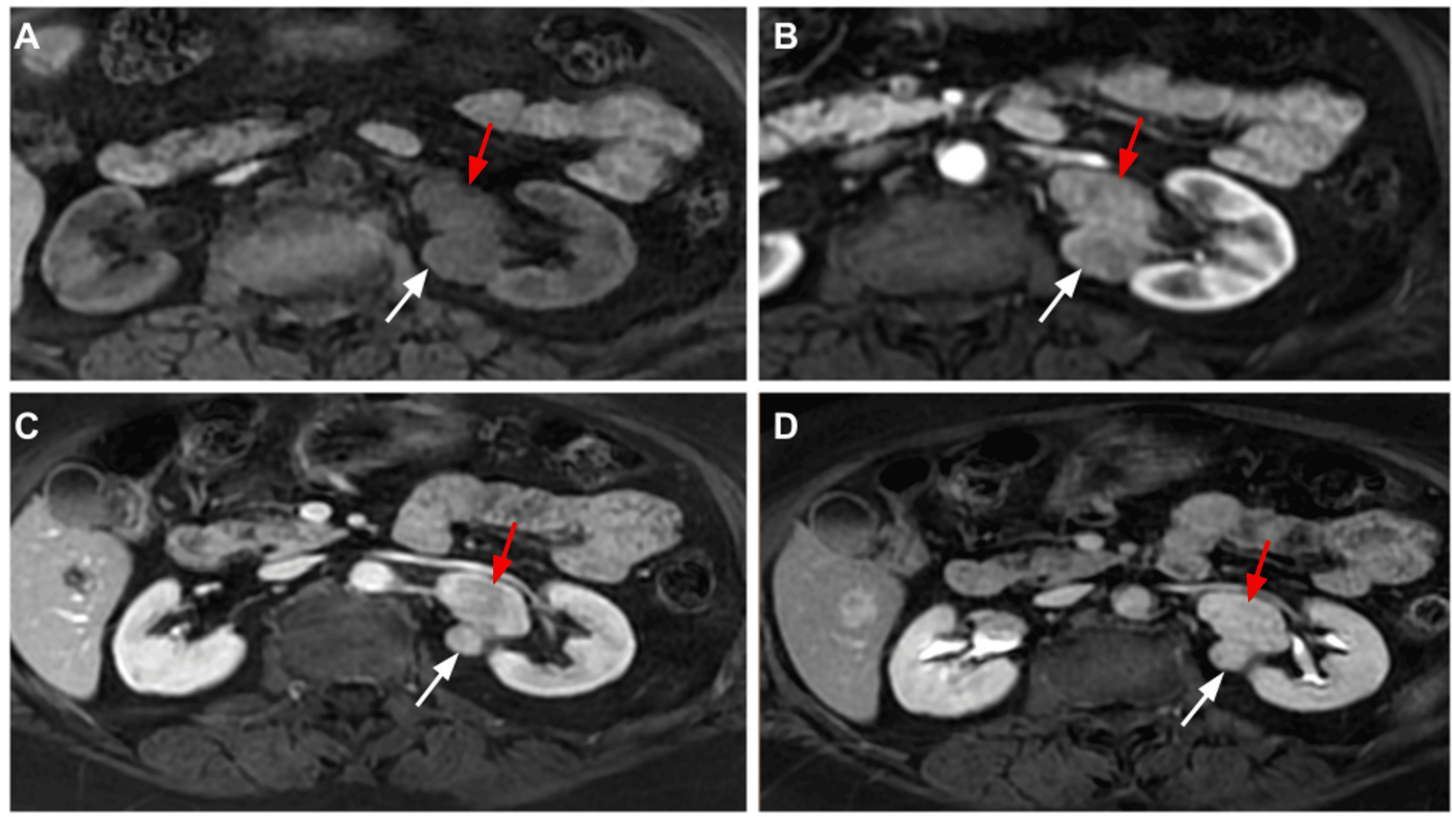
Follow up MRI abdomen three years later demonstrates relatively stable lesion that was identified previously (white arrow). And there is interval development of a new lesion within the left renal pelvis (red arrow) which has T1 intermediate signal intensity (A) and progressive enhancement of corticomedullary (B), nephrographic (C), and (D) excretory phases.

MRI confirmed a new left renal lesion which demonstrates progressive enhancement on contrast administration (Figure 3). This lesion involves the renal pedicle and is found to encase the left renal artery sparing the renal vein. Given these findings, the patient is scheduled for follow-up imaging in three to four months. If further progression is observed, a left radical nephrectomy will be conducted. Currently, the patient remains asymptomatic, with no hematuria, flank pain, or systemic symptoms such as weight loss or fever. Surveillance imaging will dictate further management, with surgical intervention planned if tumor progression continues or complications develop.

## Discussion

Peripheral nerve sheath tumors are a spectrum of benign and malignant neoplasms comprising neurofibromas, schwannomas, perineuromas, hybrid tumors, classic malignant peripheral nerve sheath tumors, epithelioid cell malignant peripheral nerve sheath tumors and perineural malignant peripheral nerve sheath tumors [6]. Most of the tumors occur sporadically while some may demonstrate association with neurocutaneous syndromes such as neurofibromatosis type 1 and 2. Our patient was diagnosed with renal schwannoma which is a relatively rare condition. Schwannomas occur secondary to extensive proliferation of myelin-sheath-forming Schwann cells and are more frequently observed along the cranial, peripheral, motor, or sympathetic nerve fibers of the head and neck region. Renal schwannomas are commonly seen arising from the hilum/pelvis (51%) followed by renal parenchyma (43%) and renal capsule (5%) [5].

Renal schwannomas tend to be more common (63%) in the female population [7]. These are slow-growing tumors and patients are typically asymptomatic. Occasionally, patients may present with malaise, fever, vague flank pain, and hematuria. On CT, schwannomas are well-defined encapsulated soft tissue lesions with homogenous or heterogenous contrast enhancement. Larger tumors may demonstrate areas of cystic or hemorrhagic degeneration which would result in tumor heterogeneity. On MRI, schwannomas have iso-to-hypointense signal on T1-weighted and hyperintense signal on T2-weighted sequences relative to the muscle. The signal intensities may vary if the tumor is large and has cystic or hemorrhagic degeneration. Variable enhancement is seen with gadolinium administration. However, the imaging findings are nonspecific in a majority of the cases, and histologic examination provides further confirmation.

Typically, the histology of schwannomas shows Antoni A and Antoni B areas. Antoni A refers to the dense arrangement of spindle cells containing palisading nuclei (Verocay bodies) [7]. On the other hand, Antoni B refers to a loose cellular arrangement with interspersed myxoid and hyaline components [7]. On imaging, the Antoni A areas correspond to the enhancing component and the Antoni B areas correspond to the non-enhancing component on contrast administration [8]. S-100 is an immunohistochemical marker specific for Schwann cells and aids in diagnosis based on the extent of positive staining. Malignant transformation of schwannomas is rare and only four malignant cases were reported in the literature [7]. Histologically, schwannoma is more likely to be malignant if it is more than 5 cm in size, contains cellular atypia or infiltrative growth, has Ki-67 of 5-65%, and has intratumoral necrosis or hemorrhage [2].

Renal schwannomas, although rare, have shown good prognosis in the reported cases [4]. Surgical resection with radical or partial nephrectomy is the definitive treatment for renal schwannomas which therefore optimizes renal function through preserving functioning renal parenchyma [5]. However, invasion of the vascular structures makes the resection challenging for the surgeons.

## Conclusions

Renal schwannomas are exceptionally rare solid renal neoplasms and often pose a diagnostic challenge due to their nonspecific clinical presentation. In this case, a 73-year-old female patient presented with an incidental renal mass which was later confirmed to be a schwannoma. Being familiar with the clinical presentation, imaging findings, and its management is crucial in prompt diagnosis and management. Although rare, the possibility of renal schwannoma should not be dismissed until further definitive confirmation with histopathology.
